# Fist‐Palm Test May Identify Mild Cognitive Impairment and Freezing of Gait in Parkinson's Disease: A Machine Learning Approach

**DOI:** 10.1002/mdc3.70080

**Published:** 2025-04-19

**Authors:** Sofia Cuoco, Michela Russo, Carlo Ricciardi, Beatrice D'Arco, Cristiano Sorrentino, Arianna Cappiello, Maria Romano, Francesco Amato, Marco Aiello, Roberto Erro, Marianna Amboni, Paolo Barone

**Affiliations:** ^1^ Department of Medicine, Surgery and Dentistry “Scuola Medica Salernitana,” University of Salerno Baronissi Italy; ^2^ Department of Electrical Engineering and Information Technology University of Naples Federico II Naples Italy; ^3^ Department of Neurology “Umberto I” Hospital Nocera Inferiore Italy; ^4^ IRCCS SynlabSDN Naples Italy

**Keywords:** cognitive deficit, FiPaT, FOG, MCI, Parkinson's disease

## Abstract

**Background:**

Mild cognitive impairment (MCI) and freezing of gait (FOG) are two common symptoms in Parkinson's disease (PD).

**Objectives:**

The objectives were to test the strength of association of fist‐palm test (FiPaT), a nonverbal motor test, with both MCI and FOG in PD and investigate the predictive ability of FiPaT in the identification of PD patients with MCI or FOG.

**Methods:**

We enrolled 74 PD patients: 47 of 74 patients had MCI (PD + MCI), 27 of 74 were cognitively unimpaired (PD‐NC), 29 of 74 presented FOG (PD + FOG), and 45 of 74 were without FOG (PD‐FOG). We performed univariate statistical analysis, including binary logistic regressions, to determine the variables that distinguish PD + MCI from PD‐NC and PD + FOG from PD‐FOG. We implemented machine learning algorithms with feature selection, using clinical‐demographic features and FiPaT scores as input variables, and the presence of MCI or FOG as features to predict.

**Results:**

FiPaT total error and topography errors (*P* = 0.049 and *P* = 0.026) significantly discriminated PD + MCI and PD‐NC, whereas disease duration (*P* = 0.039), FiPaT total error (*P* = 0.026), and attention errors (*P* = 0.046) significantly discriminated PD + FOG and PD‐FOG. Applying machine learning analysis, the models reached an accuracy of 87.4% for MCI and 78.2% for FOG.

**Conclusions:**

A worse performance on FiPaT and its subscores is closely related to MCI and FOG in PD. The early identification of MCI and FOG is of particular interest as both are important risk factors for PD dementia. Moreover, the association between FiPaT and FOG strengthens the close relationship between motor system, namely gait, and higher‐order cognitive functions.

Mild cognitive impairment (MCI) and freezing of gait (FOG) are two common symptoms in Parkinson's disease (PD).^1^ PD‐MCI,^2^ defined as a cognitive decline, not normal for age but with essentially normal functional activities, in the context of established PD, increases the risk for developing dementia (PDD).^3^, ^4^, ^5^ On the other side, FOG is a brief, episodic absence or marked reduction in forward progression of the feet despite the intention to walk, and it is a risk factor for cognitive decline in PD.^6^, ^7^, ^8^, ^9^ Understanding the association between FOG and cognition could be relevant for PD patients.^10^ In this regard, several studies have already shown that PD patients with FOG present a higher level of decline in global cognition, executive function/attention, language, memory, and visuospatial abilities than those without FOG.^7^, ^9^, ^10^, ^11^, ^12^ In addition, FOG reflects the close relationship between cognitive and motor systems,^8^, ^9^, ^10^ which is particularly evident in human and nonhuman primates during both phylogenetic and ontogenetic developments, thus testifying the continuous interactions between motor and cognitive functions.^13^, ^14^

We recently analyzed the psychometric properties of a novel nonverbal motor test, named fist‐palm test (FiPaT), which consists of two sequences of 10 trials each and is able to measure cognitive abilities.^15^ In our first study, which included patients with normal cognition and MCI (no PD), we demonstrated that FiPaT is an acceptable and reliable nonverbal test, able to screen for global cognitive status, attention and executive functions, and even to predict MCI.^15^

Based on both its easy administration and sensitivity to capture attention and executive dysfunctions, FiPaT might represent a useful screening tool for cognitive decline in PD. In addition, being a cognitive test relying on a motor task, FiPaT seems to reflect the relationship between cognitive and motor systems that underlies FOG.

Given the psychometric characteristics of the FiPaT and the relationship between cognitive and motor systems,^14^, ^15^ the main aim of the present study was twofold: (1) testing the strength of association of both FiPaT total scores and its subscores with MCI and FOG in PD and (2) investigating the predictive ability of FiPaT in the identification of PD patients with MCI or FOG. To this objective, first we used traditional statistical methods and then implemented machine learning (ML) algorithms with feature selection, using clinical‐demographic features and FiPaT scores as input variables, and the presence of MCI or FOG as features to predict.

## Methods

### Study Population

The study population consisted of 74 patients with PD, consecutively enrolled at the Center for Neurodegenerative Diseases of the University of Salerno, Italy. The diagnosis of PD was confirmed by a movement disorder specialist according to the MDS clinical diagnostic criteria for PD.^16^ All patients underwent the protocol in the ON phase.

### Materials and Methods

All enrolled patients were classified in PD patients with mild cognitive impairment (PD + MCI) or cognitively unimpaired (PD‐NC), using an extensive second‐level neuropsychological battery according to the criteria of Litvan et al.^2^ In addition, the presence of FOG was defined by obtaining a score ≥1 on the freezing question #2.13 of Part II of the Movement Disorder Society‐Unified Parkinson's Disease Rating Scale (MDS‐UPDRS). Based on these definitions, among the enrolled patients, 47 were PD + MCI and 27 were PD‐NC, whereas 29 were affected by FOG (PD + FOG) and 45 did not show FOG (PD‐FOG). Table 1 presents a contingency cross table illustrating the frequencies of MCI e FOG among the enrolled patients.

**TABLE 1:** Contingency cross table reporting the frequencies of freezing of gait (FOG) and mild cognitive impairment (MCI) in the sample

	PD + FOG	PD‐FOG	Total
PD + MCI	18	29	47
PD‐NC	11	16	27
Total	29	45	74

Abbreviations: PD + FOG, PD patients with freezing of gait; PD‐FOG, PD patients without freezing of gait; PD, Parkinson's disease; +MCI, patients with mild cognitive impairment; NC, normal cognition.

For each enrolled PD patient, the main demographic and clinical characteristics were recorded, including age, gender, education, and disease duration. The severity of motor symptoms was evaluated using the Part III of the MDS‐UPDRS.^17^ The percentage of participants showing dysfunction in each cognitive domain, that is, memory, visuospatial, attention and executive domain, and medications report are presented in Supplementary Material S1 (Tables 1 and 2).

**TABLE 2:** Comparison of clinical‐demographic features and the fist‐palm test (FiPaT) scores between PD + MCI versus PD‐NC and PF + FOG versus PD‐FOG

	PD + MCI (N = 47)	PD‐NC (N = 27)	*P*‐value
Age (Years)	72.00 ± 6.58	65.74 ± 7.39	0.021*
Gender (F/M)	17/30	5/22	0.089
Disease duration	5.75 ± 3.72	6.50 ± 6.02	0.865
Education (years)	9.26 ± 5.11	11.74 ± 4.56	0.039*
MDS‐UPDRS Part III	23.58 ± 9.40	18.43 ± 8.41	0.039*
FiPaT total error	1.96 ± 2.05	0.89 ± 1.76	0.003*
Perseverance total error	0.55 ± 0.80	0.22 ± 0.58	0.053
Attention total error	0.47 ± 0.78	0.19 ± 0.56	0.075
Planning total error	0.32 ± 0.56	0.19 ± 0.48	0.226
Topography total error	0.68 ± 0.84	0.30 ± 0.61	0.043*

	PD + FOG (N = 29)	PD‐FOG (N = 45)	*P*‐value
Age (Years)	71.24 ± 6.89	68.73 ± 7.75	0.210
Gender (F/M)	7/22	15/30	0.282
Disease duration	7.70 ± 5.02	5.09 ± 4.87	0.002*
Education	9.21 ± 4.05	10.78 ± 4.99	0.163
MDS‐UPDRS Part III	24.28 ± 9.36	18.89 ± 8.61	0.003*
FiPaT total error	2.34 ± 2.36	1.07 ± 1.57	0.008*
Perseverance total error	0.66 ± 0.86	0.29 ± 0.63	0.040*
Attention total error	0.66 ± 0.86	0.18 ± 0.53	0.003*
Planning total error	0.41 ± 0.63	0.18 ± 0.44	0.058
Topography total error	0.69 ± 0.89	0.44 ± 0.69	0.286

Abbreviations: PD, Parkinson's disease; MCI, mild cognitive impairment; NC, normal cognition; FOG, freezing of gait; MDS‐UPDRS Part III, Movement Disorder Society‐Unified Parkinson's Disease Rating Scale, Part III; FiPaT, fist‐palm test.

* Significance level at 0.05.

The entire sample underwent the FiPaT, a nonverbal test consisting of two sequences with 10 trials each. In each trial, the subjects are requested to successively place one hand over the other in two following postures: a fist resting vertically (ie, fist) and a palm resting horizontally (ie, palm). Therefore, for each of the two sequences, the subject must do the fist‐palm combination for 10 consecutive times. In the first sequence, the participants are requested to perform the task with their dominant hand, after observing the examiner complete a sequence of 10 trials. The second sequence consists of the same task but performed with the other hand (ie, with the nondominant hand and without the visual prompt provided by the examiner. During these trials, the clinician may identify four potential qualitative errors: error in topography, perseverance, attention, and planning. Specifically, error in topography represented a mistake in the spatial orientation of the gesture (for instance, the subject performed a palm trial vertically) without modifying the sequence of the trials. Perseverance was the error in which the subject repeated an item more than once. Attention error reflected the reversal of the sequence of the test (ie, the subject performed a palm‐fist sequence) or a single mistake with subsequent recovery. Finally, planning error represented initial hesitation producing wrong and/or inappropriate actions at the beginning of the sequence, subsequently followed by the correct task (for more specific information, including the scoresheet and a demonstration video, please refer to Cuoco et al.^15^. Each error was scored as 1, and the final score was the sum of all errors performed in Sequence_1 and Sequence_2 and could range from a minimum score of 0, which equated to a total correct performance, to a maximum score of 8, reflecting the presence of all error types in both sequences.^15^

### Statistical Analysis

#### Univariate Statistical Analysis

Univariate statistical analysis was performed to quantify the clinical‐demographic characteristics of the PD patients and FiPaT test scores. *IBM SPSS version 29* was used for all statistical analysis. The normality of the data was evaluated using the Shapiro–Wilk test. For normally distributed data, the Levene test was used to assess the homoscedasticity of the variances between the groups. An independent sample *t* test was performed when both the previous assumptions were verified; otherwise the Mann‐Whitney test was employed.^18^ χ^2^ test was used to determine the relationship between the two qualitative variables, FOG and MCI.

#### Binary Logistic Regression

Binary logistic regression^19^ was performed to produce models capable of classifying patients into two diagnostic groups (PD + MCI vs. PD‐NC and PF + FOG vs. PD‐FOG), considering clinical‐demographic variables and FiPaT scores. For each variable included in the models, odds ratios with 95% confidence intervals and relative *P*‐values were reported. The overall accuracy for each model was determined. Finally, the Hosmer‐Lemeshow goodness‐of‐fit test was used to demonstrate the good overall quality of the models. The alpha significance level was set at *P* < 0.05 for all statistical analyses.

#### Classification Model

A classification study was carried out using KNIME analytics platform (version 4.7.6). This platform is powerful in terms of data manipulation, for creating complex workflows, and allows users to create workflow of data analysis by combining nodes. In literature, it has been considered in various health care fields^20^ and already used in neurological studies.^21^, ^22^, ^23^

In this study, the workflow of the ML analysis was carried out using a hold‐out validation strategy; the dataset was divided into two non‐overlapping parts, and these two parts were used for training (70%) and testing (30%). In addition, a feature selection analysis was performed to define the best subset of features and build a more reliable classification model. A wrapper method based on backward feature elimination was performed to choose the main features for this analysis. In particular, the variables with the lowest significance were removed from the model.^24^ Five different classification models were employed: decision tree (DT), random forest (RF), k‐nearest neighbors (k‐NN), naïve Bayes (NB), and support vector machine (SVM). DT is a decision model that uses a tree structure of decisions and their possible consequences; RF is an extension of the DT method through ensemble learning techniques: different trees are learned in parallel on a random subset of features. k‐NN is a nonparametric algorithm that uses proximity to classify the grouping of a single data point. NB is based on Bayes' theorem. It assumes that every pair of features to be classified is independent of one another. Finally, the SVM aims to find a hyperplane in an N‐dimensional space, where N is the number of features that distinctly classifies the data points.

The main evaluation metrics were as follows:

- Accuracy (Acc) is the ratio of correct predictions over the total number of records.
- Precision (Pr) represents a measure of the positive patterns correctly predicted from the total predicted patterns in a positive class.
- Specificity (Sp) is the “true negative rate” or, in this study, the capacity to correctly detect patients not belonging to the group under examination.
- Sensitivity (Se) is the “true positive rate” or, in this study, the capacity to correctly detect patients belonging to the group under examination.

The formulas for the calculation of each metrics are presented in the Supplementary Material S1 (Table 3).

**TABLE 3:** Binary logistic regression results for PD + MCI versus PD‐NC and PD + FOG versus PD‐FOG

Variables	Odds ratio (95% CI)	*P*‐value
PD‐MCI vs. PD‐NC		
Age (years)	NI	NI
Disease duration	NI	NI
Education	NI	NI
MDS‐UPDRS Part III	0.922 (0.870–0.997)	0.087
FiPaT total error	1.318 (1.152–1.654)	0.049*
Perseverance total error	NI	NI
Attention total error	NI	NI
Planning total error	NI	NI
Topography total error	1.318 (1.202–1.664)	0.026*
PD + FOG vs. PD‐FOG		
Age (years)	NI	NI
Disease duration	0.922 (0.874–0.973)	0.039*
Education	NI	NI
MDS‐UPDRS Part III	1.318 (1.172–1.854)	0.054
FiPaT total error	1.318 (1.174–1.857)	0.026*
Perseverance total error	0.961 (0.852–0.996)	0.104
Attention total error	1541 (1.324–1.647)	0.046*
Planning total error	1115 (1.144–1.457)	0.063
Topography total error	NI	NI

Note: Odds ratio (95% confidence interval) and *P*‐value for each variable were considered in the model.

Abbreviations: CI, confidence interval; PD, Parkinson's disease; MCI, mild cognitive impairment; NC, normal cognition; FOG, freezing of gait; MDS‐UPDRS Part III, Movement Disorder Society‐Unified Parkinson's Disease Rating Scale, Part III; FiPaT, fist‐palm test; NI, not included.

* Significance level at 0.05.

## Results

When analyzing the relationship between FOG and MCI using χ^2^ test, no significant difference was observed (*P*‐value = 0.524). Univariate statistical analysis, binary logistic regression, and ML analysis were performed twice. First, the analysis aimed to compare and distinguish PD + MCI (*N* = 47) from PD‐NC (N = 27). Subsequently, the analysis was conducted to confront and discriminate PD + FOG (N = 29) from PD‐FOG (N = 45).

### Statistical Analysis

The univariate statistical analysis comparing clinical‐demographic features and FiPaT test scores between PD + MCI and PD‐NC and between PD + FOG and PD‐FOG patients is presented in Table 2.

PD + MCI patients were older, less educated, and with more severe motor symptoms compared to PD‐NC patients, as indicated by increased MDS‐UPDRS III. In addition, PD + MCI patients reported higher scores on both FiPaT total error and topography total error compared to PD‐NC patients, whereas PD + FOG patients exhibited longer disease duration and more severe motor symptoms than PD‐FOG patients. Furthermore, PD + FOG exhibited higher scores on FiPaT total error, perseverance total error, and attention total error than PD‐FOG.

## Binary Logistic Regression

Binary logistic regression was performed to distinguish diagnostic groups (PD + MCI vs. PD‐NC and PF + FOG vs. PD‐FOG). The results are presented in Table 3.

Two outliers were removed from the comparison between PD + MCI and PD‐NC, and other two outliers were removed from the analysis between PD + FOG and PD‐FOG, based on diagnostic measures such as Cook's distance.

A backward selection process, which retained only those predictors showing a potential relevance in the context of the analysis, was used to build the binary logistic regression.

Regarding PD + MCI versus PD‐NC, three variables were included in the model: MDS‐UPDRS Part III, FiPaT total error, and topography total error; among these, FiPaT total error and topography total error resulted statistically significant (*P*‐value = 0.049 and *P*‐value = 0.026, respectively), whereas MDS‐UPDRS Part III showed a statistical trend (*P*‐value = 0.083). Regarding PD + FOG versus PD‐FOG, six variables were included in the model, of which three variables were statistically significant, namely disease duration (*P*‐value = 0.039), FiPaT total error (*P*‐value = 0.026), and attention total error (*P*‐value = 0.046), whereas MDS‐UPDRS Part III and planning total error showed a statistical trend (*P*‐value = 0.054 and *P*‐value = 0.063, respectively).

Table 4 shows the confusion matrix of each model. The overall accuracies of the models were 76.4% and 72.2% for MCI and FOG, respectively. The ability to detect PD patients affected by a specific symptom was 84.1% for MCI and 73.0% for FOG. The odds ratios of the variables included in the MCI model demonstrated that a higher FiPaT total error and topography total error scores were associated with a higher likelihood of being in the PD + MCI group. As with the FOG model, higher FiPaT total error, planning total error, attention total error, and higher MDS‐UPDRS Part III scores were associated with a higher likelihood of being in the PD + FOG group. The Hosmer‐Lemeshow test showed a *P*‐value of 0.145 and 0.219 for the MCI and FOG models, respectively. For this test, a *P*‐value >0.05 indicates that there are no significant differences between the observed and predicted frequencies.

**TABLE 4:** Confusion matrix of binary logistic regression for PD + MCI versus PD‐NC and PD + FOG versus PD‐FOG

		Predicted		%
		PD + MCI	PD‐NC	
Observed	PD + MCI	37	10	
	PD‐NC	7	18	
	%	84.1	64.3	76.4

		Predicted		
		PD + FOG	PD‐FOG	%
Observed	PD + FOG	19	10	
	PD‐FOG	10	33	
	%	65.5	76.7	72.2

Abbreviations: PD, Parkinson's disease; MCI, mild cognitive impairment; NC, normal cognition; FOG, freezing of gait.

### Classification Analysis

The ML analysis was performed twice: first, it was performed on MCI and then on FOG. The classification results are presented in Table 5.

**TABLE 5:** Evaluation metrics (%) of the machine learning (ML) algorithms computed between PD + MCI versus PD‐NC and PD + FOG versus PD‐FOG

Classifiers	Features selected	Accuracy	Sensitivity	Specificity	Precision
PD + MCI vs. PD‐NC					
SVM	MDS‐UPDRS Part III FiPaT total error Topography total error	65.3	77.8	51.5	67.7
NB	MDS‐UPDRS Part III Topography total error Attention total error	83.2	57.4	94.2	83.6
k‐NN	MDS‐UPDRS Part III FiPaT total error Topography total error	87.4	75.5	93.4	86.4
DT	MDS‐UPDRS Part III Planning total error Attention total error	74.1	82.3	67.3	80.1
RF	Education FiPaT total error Attention total error	83.6	75.1	87.1	87.2
PD + FOG vs. PD‐FOG					
SVM	Disease duration Education Attention total error	65.1	41.0	87.0	64.1
NB	FiPaT total error Topography total error Attention total error	78.2	24.0	89.2	73.3
k‐NN	Disease duration MDS‐UPDRS Part III Attention total error	73.4	55.3	91.3	83.5
DT	FiPaT total error Perseverance total error Attention total error	74.4	67.6	82.6	80.0
RF	FiPaT total error Topography total error Attention total error	73.2	56.4	87.0	75.4

Note: Features selected by each algorithm using backward feature elimination.

Abbreviations: PD, Parkinson's disease; MCI, mild cognitive impairment; NC, normal cognition; FOG, freezing of gait; MDS‐UPDRS Part III, Movement Disorder Society‐Unified Parkinson's Disease Rating Scale, Part III; FiPaT, fist‐palm test; SVM, support vector machine; NB, naïve Bayes; k‐NN, k‐nearest neighbors; DT, decision tree; RF, random forest.

The backward feature elimination selected three main features for each individual classifier. Particularly, for the MCI model, the most recurrent features were MDS‐UPDRS Part III, with a frequency of 80%, FiPaT, attention, and topography total error, with a frequency of 60%. On these selected features, the best results were obtained by k‐NN, which showed the higher Acc (87.4%) and the best trade‐off among all evaluation metrics (Sp = 93.4%, Se = 75.5%, and Pr = 86.4%), and RF, which reached the highest Pr (87.2%). On the FOG model, the most recurrent features were attention total error, with a frequency of 100%, FiPaT total error, with a frequency of 60%, topography total error followed by disease duration. The highest accuracy was achieved by NB (78.2%), followed by DT, which showed the highest Se (67.6%); k‐NN demonstrated the highest Sp (91.3%) and Pr (83.5%).

## Discussion

In the present study, we assessed the ability of FiPaT, a bedside motor tool for screening global cognitive status, to identify MCI and FOG in a sample of patients with PD, using traditional statistical analysis and ML procedures.

First, we found that PD‐MCI patients were significantly older, less educated, and had greater motor disabilities than PD‐NC; they also scored worse on FiPaT, with significantly higher errors in topography compared to PD‐NC. The association between FiPaT total score and its subscore topography errors with the presence of PD‐MCI was further confirmed by regression analysis, which achieved an accuracy of 76.4%, and, subsequently, by the most robust ML models. In fact, according to k‐NN model, FiPaT total score, errors in topography, and MDS‐UPDRS Part III resulted in the most relevant variables to identify MCI in PD, with a high level of accuracy, sensibility, and specificity. Of note, FiPaT total score, or at least one of its error categories, was always selected in any model. Our findings are in line with the literature of PD showing that MCI is often associated with old age, low education, and more severe motor dysfunction.^25^ In addition, data regarding FiPaT are perfectly in line with our previous study in which patients with MCI showed a worse score than NC. Moreover, in the previous study, we found that both FiPaT and Montreal Cognitive Assessment (MoCA) scores were significant predictors of MCI, and that errors in topography were the most significant in patients with MCI with selective attentional domain disorder, which is, indeed, a common presentation of PD‐MCI.^15^, ^26^ Consistently, in the present study, among all error categories, the topographical one represents the most common selected category for the identification of PD‐MCI. In fact, it was chosen by both the regression analysis and several ML models. We hypothesize that errors in topography may mirror the spreading of cognitive dysfunction toward more posterior brain areas, namely temporo‐parieto‐occipital cortices. Indeed, error in topography relies on altered integration between short‐term memory storage of a “copy” of the various representational steps, mental representation, and monitoring ability.^27^, ^28^, ^29^

Regarding FOG, we found that PD patients with FOG presented longer disease duration, more severe motor symptoms, increased FiPaT total score, and higher errors of perseverance and attention than PD without FOG. The clinical findings are in line with previous literature reporting that FOG is associated with more severe motor symptoms and its occurrence increases as the disease progresses.^30^, ^31^ As to FiPaT findings, perseverative and attentional errors typically are expression of dysexecutive and attentional deficits that are usually neuropsychological core features of PD patients with FOG.^12^ The relationship of FOG with disease duration, FiPaT total score, and attention total errors was further confirmed using regression analysis. Interestingly, attention total errors category represents also the feature selected by all ML models, thus supporting a strict association between attentional dysfunction and FOG.^32^, ^33^, ^34^ Moreover, clinical variables, such as disease duration and/or MDS‐UPDRS Part III, have been selected only in two (SVM and k‐NN) out the five models, whereas FiPaT total score or at least one of its error categories was included in all models, strengthening again the role of cognitive dysfunction in FOG.

Based on the actual knowledge on the structural and functional changes associated with FOG, further speculations can be hypothesized on our findings suggesting a close relationship between FiPaT and FOG. Specifically, as reported by Fasano et al,^35^ FOG appears to be partly related to disruptions in the “executive attention” network along with regional tissue loss in widespread cortical areas, including the premotor area, inferior frontal gyrus, precentral gyrus, parietal, and occipital areas, the latter being involved in visuospatial processing. Likewise, we hypothesized that the proper execution of FiPaT requires the integrity and the interactions between frontal and posterior brain areas for the correct processing and monitoring of a motor plan based on visuospatial input. Accordingly, FiPaT could be considered a sort of proxy of the relationship between cognitive and motor systems, as its execution should theoretically engage large‐scale cortical networks, involving temporal, parietal, and frontal areas, that appear to play a primary role in controlling different aspects of motor and cognitive functions.^14^ However, further studies are needed to support our hypothesis regarding the brain networks engaged in the execution of FiPaT.

The present study has some limitations. First, the relatively small sample size and the lack of reproducibility of the test in a nonspecialized setting and/or on healthy controls might limit the generalizability of the results. Second, the absence of subtyping FOG based on levodopa responsiveness (responsive, unresponsive, drug‐induced) could have affected some findings. Third, the presence of FOG was assessed through self‐reporting. It is well known that clinical assessment of FOG may be quite challenging due to its episodic and unpredictable nature, particularly during a routine motor examination when patients commonly tend to focus their attentional resources on walking, thus switching from automatic to voluntary control of locomotion.^36^, ^37^ Actually, recent studies showed that some gait tasks might represent reliable and valid tools to identify patients with FOG,^38^, ^39^ although, as the authors themselves admit, such FOG‐provoking tasks may increase specificity of self‐reporting FOG rather than replace it.^39^ Finally, we did not evaluate the patients' performance on FiPaT in OFF medications and the potential Alzheimer co‐pathology by means of fluid biomarkers dosing, that is, Aβ 42 and specific *p*‐tau.

Despite these limitations, our preliminary findings suggest that worse performance on FiPaT and its subscores is closely related with MCI and FOG in PD. We predict that longitudinal studies might demonstrate that FiPaT may represent a valid, bedside screening tool for identifying PD patients at risk of developing MCI and/or FOG. The early identification of MCI and FOG is of particular interest as both of them are important risk factors for PDD.^31^, ^40^ Finally, further prospective studies on a nonselected cohort could strengthen the association between FiPaT and FOG, thus contributing to the hypothesis that there is a connection between walking, gestures, and higher‐order cognitive functions.^13^, ^14^

## Author Roles

(1) Research project: A. Conception, B. Organization, C. Execution; (2) Statistical analysis: A. Design, B. Execution, C. Review and critique; (3) Manuscript preparation: A. Writing of the first draft, B. Review and critique.

S.C: 1B, 1C, 3C, 3A

M.R: 2A, 2B, 3C

C.R: 2A, 2B, 3C, 3B

B.D.A: 1C, 3B

C.S: 1C, 3B

A.C: 3B

M.R: 3B

F.A: 3B

M.A: 3B

R.E: 1C, 3B

M.A: 1A, 2C, 3B

P.B: 1A, 2B, 2C, 2B

## Disclosures

**Ethical Compliance Statement:** The project was performed in accordance with the ethical standards laid down in the 1964 Declaration of Helsinki. All subjects agreed with a written consent form to the use of their anonymized data for research purposes, the study being approved by the local ethical committee. We confirm that we have read the journal's position on issues involved in ethical publication and affirm that this work is consistent with those guidelines.

**Funding Sources and Conflict of Interest:** No specific funding was received for this work. The authors declare that there are no conflicts of interest relevant to this work.

**Financial Disclosures for the Previous 12 months:** P.B. received consultancies as a member of the advisory board for Zambon, Lundbeck, UCB, Chiesi, Abbvie, and Acorda; R.E. receives royalties from publication of Case Studies in Movement Disorders—Common and Uncommon Presentations (Cambridge University Press, 2017) and of Paroxysmal Movement Disorders (Springer, 2020). He has received consultancies from Ipsen and honoraria for speaking from the International Parkinson's Disease and Movement Disorders Society. The other authors report no financial disclosures.

## Supporting information


**Table S1.** Percentage of participants showing dysfunction per each cognitive domain.
**Table S2.** Medications data. LED, levodopa equivalent dose; MAO‐B, monoamine oxidase‐B; COMT, catechol‐*O*‐methyltransferase; LEDD, levodopa equivalent daily dose; N.A, not applicable.
**Table S3.** Confusion matrix and subsequent computation of sensitivity, specificity, accuracy, and precision. FN, false negative; FP, false positive; TN, true negative; TP, true positive.

## Data Availability

The data that support the findings of this study are available from the corresponding author upon reasonable request.
