# Supplementary material for: Fist‐Palm Test May Identify Mild Cognitive Impairment and Freezing of Gait in Parkinson's Disease: A Machine Learning Approach
Source: Mov Disord Clin Pract. 2025 Apr 19;12(8):1105–12. doi: 10.1002/mdc3.70080 (PMC12371445; doi:10.1002/mdc3.70080)
Supplement: Supplementary file 1 — Table S1. Percentage of participants showing dysfunction per each cognitive domain. Table S2. Medications data. LED, levodopa equivalent dose; MAO‐B, monoamine oxidase‐B; COMT, catechol‐O‐methyltransferase; LEDD, levodopa equivalent daily dose; N.A, not applicable. Table S3. Confusion matrix and subsequent computation of sensitivity, specificity, accuracy, and precision. FN, false negative; FP, false positive; TN, true negative; TP, true positive. [file MDC3-12-1105-s001.docx]

**Supplementary Material**

**Table 1:** Percentage of participants showing dysfunction per each cognitive domain

| **Cognitive data** | **Compromise percentage** |
| --- | --- |
| **Memory domain** | 31.08% |
| **Visuo-spatial domain** | 24.32% |
| **Attentional domain** | 33.78% |
| **Executive domain** | 43.24% |

**Table 2:** Medications data

| **Medications** | **Mean ± standard deviation (mg)** | **Usage percentage** |
| --- | --- | --- |
| **Levodopa daily dose** | 465.49±249.67 | 68.91% |
| **Dopamine agonists LED** | 165.58±91.19 | 32.43% |
| **MAO-B Inhibitors LED** | 102.08±10.20 | 32.43% |
| **COMT Inhibitors LED** | 188.57±106.05 | 9.45% |
| **Amantadine LED** | 100.00±0.00 | 2.70% |
| **LEDD** | 546.27±316.16 | N.A. |

**Abbreviations:** LED: Levodopa equivalent dose; MAO-B: monoamine oxidase-B; COMT: catechol-O-methyltransferase; LEDD: levodopa equivalent daily dose; N.A.: Not Applicable

**Table 3.** Confusion matrix and subsequent computation of Sensitivity, Specificity, Accuracy and Precision

|  | | Actual Values | |  |
| --- | --- | --- | --- | --- |
|  |  | Positive | Negative |  |
| Predicted Values | Positive | TP | FP | Precision  $\frac{TP}{(TP+FP)}$ |
|  | Negative | FN | TN |  |
|  |  | Sensitivity  $\frac{TP}{(TP+FN)}$ | Specificity $\frac{TN}{(TN+FP)}$ | Accuracy  $\frac{TP+TN}{(TP+TN+FP+FN)}$ |

**Abbreviations**: FN, False Negative; FP, False Positive; TN, True Negative; TP, True Positive.
